# Pre-exposure prophylaxis for HIV-negative persons with partners living with HIV: uptake, use, and effectiveness in an open-label demonstration project in East Africa

**DOI:** 10.12688/gatesopenres.12752.2

**Published:** 2018-01-30

**Authors:** Renee Heffron, Kenneth Ngure, Josephine Odoyo, Nulu Bulya, Edna Tindimwebwa, Ting Hong, Lara Kidoguchi, Deborah Donnell, Nelly R. Mugo, Elizabeth A. Bukusi, Elly Katabira, Stephen Asiimwe, Jennifer Morton, Susan Morrison, Harald Haugen, Andrew Mujugira, Jessica E. Haberer, Norma C. Ware, Monique A. Wyatt, Mark A. Marzinke, Lisa M. Frenkel, Connie Celum, Jared M. Baeten, The Partners Demonstration Project Team

**Affiliations:** 1 Department of Epidemiology, University of Washington, Seattle, USA; 2 Department of Global Health, University of Washington, Seattle, USA; 3 College of Health Sciences, Jomo Kenyatta University of Agriculture and Technology, Nairobi, Kenya; 4 Centres for Microbiology Research , Kenya Medical Research Institute, Nairobi, Kenya; 5 Infectious Diseases Institute, Makerere University, Kampala, Uganda; 6 Kabwohe Clinical Research Center, Kabwohe, Uganda; 7 Vaccine and Infectious Disease Division, Fred Hutchinson Cancer Research Center, Seattle, USA; 8 Centres for Clinical Research, Kenya Medical Research Institute, Nairobi, Kenya; 9 Department of Obstetrics and Gynecology, University of Washington, Seattle, USA; 10 Harvard Medical School - Massachusetts General Hospital, Boston, USA; 11 Department of Global Health and Social Medicine, Harvard Medical School, Boston, USA; 12 Harvard Global, Cambridge, USA; 13 Department of Medicine, Johns Hopkins University, Baltimore, USA; 14 Seattle Children’s Research Center, Seattle, USA; 15 Department of Laboratory Medicine, University of Washington, Seattle, USA; 16 Department of Pediatrics, University of Washington, Seattle, USA; 17 Department of Medicine, University of Washington, Seattle, USA

**Keywords:** HIV prevention, HIV serodiscordant couples, PrEP, ART

## Abstract

**Background**: Pre-exposure prophylaxis (PrEP) can provide high protection against HIV infection and is a recommended intervention for HIV-negative persons with substantial HIV risk.  Demonstration projects conducted in diverse settings worldwide illustrate practical examples of how PrEP can be delivered. This manuscript presents estimates of effectiveness and patterns of PrEP use within a two-year demonstration project of PrEP for HIV-negative members of heterosexual HIV serodiscordant couples in East Africa.

**Methods**: The PrEP delivery model integrated PrEP into HIV treatment services, prioritizing PrEP use for HIV-negative partners within serodiscordant couples before and during the first 6 months after the partner living with HIV initiated antiretroviral therapy (ART).  We measured PrEP uptake through pharmacy records and adherence to PrEP through medication event monitoring system (MEMS) bottle caps and quantification of tenofovir in plasma among a random sample of participants. We estimated HIV infections prevented using a counterfactual cohort simulated from the placebo arm of a previous PrEP clinical trial.

**Results**: We enrolled 1,010 HIV serodiscordant couples that were naïve to ART and PrEP.  Ninety-seven percent of HIV-negative partners initiated PrEP. Objective measures suggest high adherence: 71% of HIV-negative participants took ≥80% of expected doses, as recorded via MEMS, and 81% of plasma samples had tenofovir detected.  Four incident HIV infections were observed (incidence rate=0.24 per 100 person-years), a 95% reduction (95% CI 86-98%, p<0.0001) in HIV incidence, relative to estimated HIV incidence for the population in the absence of PrEP integrated into HIV treatment services.

**Conclusions**: PrEP uptake and adherence were high and incident HIV was rare in this PrEP demonstration project for African HIV-negative individuals whose partners were known to be living with HIV.  Delivery of PrEP to HIV-negative partners within HIV serodiscordant couples was feasible and should be prioritized for wide-scale implementation.

## Introduction

Pre-exposure prophylaxis (PrEP) is a new intervention to contribute to control of the global HIV epidemic ^1– 4^. Delivery systems for PrEP that maximize impact and sustainability, while minimizing cost are ideal, especially in settings with limited resources and large numbers of people with substantial risk for HIV. Approaches that synergize with existing health programs – including HIV treatment, family planning, HIV testing and counseling, and antenatal care – can capitalize on opportunities that come with existing infrastructure and community health-seeking behavior, easing the process for introducing PrEP.

Demonstration projects are needed to introduce PrEP in different settings, target individuals with different levels of HIV risk, and pilot PrEP integration into different public health programs. Through demonstration projects, implementers can gauge the infrastructure needed to provide and scale up an intervention and how individuals incorporate a new intervention into their life ^5^. They can also identify populations which will easily adopt a new intervention and which populations need targeted demand creation tools. For PrEP, demonstration projects were initiated immediately following clinical trials and continue to identify models that maximize adherence and create demand among people who are the best candidates ^6^.

Partnerships between HIV-negative persons and people living with HIV, i.e. serodiscordant couples, have a high risk for transmission in the absence of HIV prevention interventions and are thus a priority population for delivery of novel HIV prevention tools. Because HIV transmission risk is greatest prior to initiation of antiretroviral therapy (ART) and consequent viral suppression in the partner living with HIV, PrEP can be a time-limited intervention for periods without ART use and/or viral suppression. Furthermore, when PrEP and ART are offered together as components of combination HIV prevention programs, couples have multiple options and can be encouraged to adopt strategies based on their preferences and true risk factors, in addition to continuous encouragement for sustained ART use by the partner living with HIV. 

We conducted a demonstration project of PrEP for HIV prevention among HIV serodiscordant couples attending four HIV care clinics in East Africa. Interim results, focusing on HIV incidence reduction from this project, were previously reported ^7^. Here, we present the final results from two years of follow up, including estimates of intervention effectiveness and patterns of PrEP uptake and use.

## Methods

*Study design.* The Partners Demonstration Project (clinicaltrials.gov #NCT02775929) was an open-label evaluation of integrated delivery of PrEP and ART for high risk HIV serodiscordant couples. Four clinics located in Kampala and Kabwohe in Uganda and Thika and Kisumu in Kenya were engaged to deliver the intervention; all clinics were HIV care centers and also had experience with HIV prevention research. Whenever possible, operations for the intervention were designed to mirror implementation strategies used in public clinics, such as the use of text messages to remind participants about clinic appointments, so as to develop a scalable delivery approach.

Each clinic recruited HIV serodiscordant couples through referrals from voluntary counseling and testing centers, antenatal clinics, and ART clinics, and by conducting community outreach events that promoted couples-based HIV testing. Eligible couples were ≥18 years of age, sexually active, and intending to remain as a couple for at least one year. At the time of enrollment, HIV-negative partners had never used PrEP, had normal renal function (defined as an estimated creatinine clearance ≥60 mL/min using the Cockcroft-Gault equation with ideal body weight), were not infected with hepatitis B virus, and were not pregnant or breastfeeding. At enrollment, HIV-positive partners were not using ART and couples were excluded if the HIV-positive partner had WHO stage III or IV HIV disease conditions that indicated immediate need for ART. In addition, an explicit goal of the project was to recruit couples at high risk of HIV acquisition, in order to demonstrate PrEP delivery in couples most likely to benefit from the intervention. For that reason, couples were eligible only if they scored at least 5 points on a validated, empiric risk scoring tool which included variables for: age of the HIV-negative partner, marital status of the couple, any condomless sex within the couple during the past 30 days, male circumcision status of the HIV-negative partner, and HIV viral load of the partner living with HIV ^8, 9^. Couples were encouraged to attend study visits together, scheduled 1 month after enrollment, 2 months later, and every 3 months thereafter for up to 24 months. At all visits, couples were offered comprehensive couples-based HIV prevention counseling, including condoms and syndromic management of sexually transmitted infections.

*PrEP delivery.* At enrollment, co-formulated emtricitabine/tenofovir disoproxil fumarate (FTC/TDF) was offered to all HIV-negative participants as PrEP with a daily dosing schedule; participants electing not to initiate PrEP at enrollment were offered PrEP initiation at subsequent visits. PrEP was prescribed and dispensed at each study visit with participants being given 1, 2, or 3 bottles corresponding to the time until their next appointment date. Adherence counseling for PrEP was conducted using streamlined messages with a focus on individual barriers to daily use and methods to overcome barriers ^10^. For participants using PrEP, serum creatinine was measured 1 month after initiation and every 6 months thereafter. Creatinine confirmatory testing was conducted in cases of a Grade 1 or higher elevation. PrEP was temporarily withheld pending confirmatory testing if the creatinine elevation was Grade 2 or higher. PrEP was permanently discontinued for confirmed Grade 2 or higher events or if creatinine clearance was <50mL/min.

Partners living with HIV were encouraged to initiate ART as soon as possible according to national guidelines. At the start of the study, ART was available in public clinics to people living with HIV whose CD4 count was <350 cells/uL or with clinical indication. Partway through the study, national guidelines in each country were revised to encourage ART initiation for any individual living with HIV in an HIV serodiscordant relationship. Thus, at that point, all partners living with HIV in this study became ART eligible. ART was available to participants through public clinics and at the demonstration project clinics. Participants living with HIV were evaluated for CD4 count and HIV RNA at study enrollment and at 6-monthly intervals during follow up.

**Figure 1.**
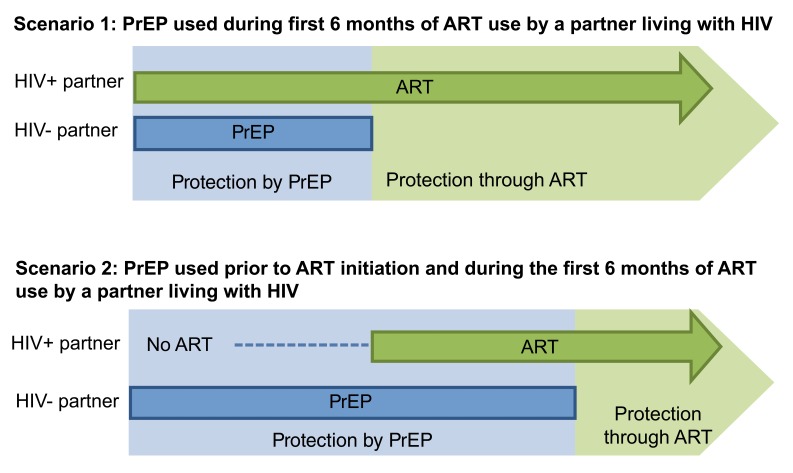
Strategy for PrEP delivery to HIV-negative persons with a partner known to be living with HIV.

HIV-negative participants using PrEP were encouraged to discontinue PrEP once their study partner living with HIV used ART for at least 6 months ( Figure 1). Results from HIV RNA testing were not required to guide counseling about PrEP use and potential viral suppression to reflect the public delivery model in which HIV RNA testing was often unavailable. If available when PrEP discontinuation was considered, HIV RNA results could be used to identify the potential for non-adherence to ART; however, testing was conducted on a 6-monthly schedule timed to study start, not the ART initiation schedule, and testing may not have aligned with ART initiation. In lieu or requiring HIV RNA results, we adopted a conservative time of 6 months of ART use to obtain viral suppression based on data from HIV serodiscordant couples in clinical trials with carefully measured HIV RNA trajectories following ART initiation ^11^. The approach of using a calendar period since ART initiation is scalable in public clinics regardless of whether HIV RNA testing becomes more available.

 This strategy of time-limited PrEP use by the HIV-negative partner until the partner living with HIV sustained 6 months of ART use was included in counseling discussions with couples beginning with study screening. In cases of ART non-adherence, new sexual partners with unknown HIV status or ART use disclosed by the HIV-negative partner, or pregnancy intentions within the couple, counselors encouraged the HIV-negative partner to continue PrEP.

*PrEP adherence.* We used medication event monitoring system (MEMS) caps to record all openings of PrEP pill bottles as our primary measure of adherence and self-reported use and pharmacy pill counts as additional measures of adherence; these were collected from all participants at all visits. Additionally, archived plasma from quarterly visits following PrEP dispensation was tested for quantification of tenofovir (TFV), the metabolized form of TDF, in a 15% random sample of participants. TFV levels were quantified using ultra-performance liquid chromatographic-tandem mass spectrometric (LC-MS/MS), with a limit of quantification of 0.31 ng/mL ^12^.

*Incident HIV infection.* HIV-negative partners underwent HIV testing prior to PrEP dispensing at each follow-up visit, following the national HIV rapid testing algorithms for Kenya and Uganda. Reactive results from rapid tests were confirmed with enzyme immunoassay (EIA) and HIV RNA quantification. For confirmed seroconverters, enrollment samples were quantified for HIV RNA; those positive for HIV RNA at enrollment were determined to have been infected prior to entry into the project. Seroconverters with negative HIV RNA results from enrollment samples were determined to have incident infections. For all seroconverters, archived plasma samples from the time point when seroconversion was detected underwent standard consensus sequencing of the *pol* region to detect HIV resistance. Additionally, for incident seroconversions, a sample from the study partner living with HIV was sequenced and sequences from both partners underwent phylogenetic analysis and posterior probability of linkage with the use of pairwise nucleotide distances between sequences to determine whether the incident infection likely originated with the study partner.

### Ethical statement

The study protocol was approved by the Human Subjects Division at the University of Washington (#STUDY00001674) and Ethics Review Committees overseeing each study site: Scientific Ethics Review Unit at the Kenya Medical Research Institute (SSC No. 2441), the Ethics Review Committee of Kenyatta National Hospital (P286/05/2012), and the AIDS Research Committee of the Uganda National Council of Science and Technology (ARC 135 and ARC126). All participants provided written informed consent.

*Statistical methods.* Characteristics of couples and patterns of PrEP use were summarized using descriptive statistics. Methods for developing a counterfactual comparison cohort have been described previously ^7^. Briefly, we used data from the placebo arm of the prior Partners PrEP Study ^1^, a PrEP clinical trial conducted from 2008–2011 among HIV serodiscordant couples in the same research clinics, to simulate a comparable “non-intervention” cohort, frequency matched to the Partners Demonstration Project by HIV risk score and duration of study follow up. The mean number of HIV infections expected in the counterfactual population was averaged over 10,000 bootstrap samples and a 95% confidence interval was defined by the 2.5 ^th^ and 97.5 ^th^ quantiles. The incidence rate ratio was computed comparing actual HIV incidence observed in the current study to the mean estimate from the counterfactual population; a 95% confidence interval was calculated using a Poisson distribution, and the p-value was estimated from the bootstrap distribution. Additional bootstrap distributions were constructed with restriction to the age and gender of the HIV-negative partner (one per gender) and enrollment plasma HIV RNA concentrations of the partner living with HIV, to create stratified estimates for these subgroups. All simulations excluded data from couples (n=3) whose HIV-infected partner was determined retrospectively to have been using ART at study enrollment. The bootstrap analyses and descriptive statistics were conducted using SAS version 9.4 (SAS Institute) and figures were generated using Powerpoint 2016 (Microsoft, USA) and Tableau 10.3 (Tableau Software, Seattle, USA).

## Results

*Participant characteristics.* In total, 1694 couples were screened and 1013 enrolled between November 2012 and August 2014. Participant visits were conducted through June 2016 when all had been followed for 24 months. Primary reasons for not being eligible to enroll included having a risk score <5, WHO stage >2 for the partner living with HIV, and abnormal renal values for the HIV-negative partner ^7^. Three couples were excluded from the analysis due to retrospectively ascertained use of ART at enrollment. At baseline, a substantial proportion of couples had characteristics that were consistent with having high risk for HIV transmission: 41% of the partners living with HIV had plasma HIV RNA concentrations >50,000 copies/ml and 65% of couples reported condomless sex in the prior month ( Table 1). Two-thirds of couples had HIV-negative male partners and 67% of these were not circumcised. Most couples were married (94%) and although the median time living together was 2.3 years (interquartile range [IQR] 0.8–6.3), very few couples had known their HIV discordant status more than a few months (median time known discordant 0.1 years, interquartile range [IQR] 0.1–0.3). Couples contributed a total of 1690.5 person-years of follow up time. Retention was above 83% at all visits. Nine hundred eighty-two (97%) of HIV-negative participants initiated PrEP, including 960 (95%) that initiated at enrollment into the project. Among women, 96.6% initiated PrEP and among men, 98.5% initiated PrEP. The median duration of PrEP use was 12 months (IQR 6-18) and condomless sex was reported at 41.2% of visits when sex with the study partner was reported.

**Table 1.** Characteristics of couples enrolled in the Partners Demonstration Project.

Characteristics of couples	N (%) or Median (IQR)
Total couples	1010
Married to each other	954 (94%)
Years living together	2.3 (0.8, 6.3)
Years aware of discordant status	0.1 (0.1, 0.3)
Proportion without children	570 (56%)
HIV risk score *	6 (6, 8)
Number of sex acts between partners, prior month **	5 (3, 10)
Any condomless sex acts between partners, prior month **	653 (65%)
Either partner had outside partners, prior month	118 (12%)
Characteristics of HIV-negative partners	
Male gender	677 (67%)
Age, years	30 (26, 36)
Education, years	8 (7, 12)
Any monthly income	868 (86%)
Circumcised, for male HIV-negative partners	452 (67%)
Characteristics of partners living with HIV	
Male gender	333 (33%)
Age, years	28 (23, 35)
Education, years	8 (6, 11)
Any monthly income	736 (73%)
CD4 cell count (cells/uL)	436.0 (272.5, 638.0)
Viral load (log _10_ copies/ml)	4.6 (3.9, 5.0)
Viral load >50,000 copies/ml	417 (41%)

*Score is out of a total possible 10 points; based on characteristics of age, marital status, children, circumcision status of negative male partner, and HIV viral load of partner living with HIV **as reported by the HIV-negative partner

**Figure 2.**
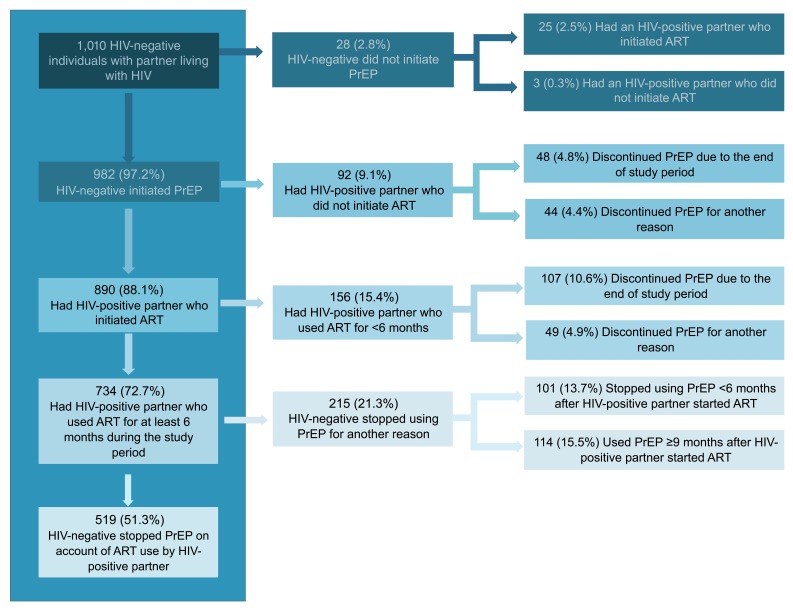
PrEP Use and Discontinuation by HIV-negative individuals with partners known to be living with HIV.

*Patterns of PrEP use and discontinuation.* Overall, 88.1% of HIV-negative partners used PrEP until their partner living with HIV was using ART, including 51.3% who used PrEP for exactly 6 months following ART initiation by their partner ( Figure 2). PrEP use for >6 months following ART initiation (by 114, 15.5% of PrEP users with at least 6 months of ART by the partner) was due to primarily to immediate fertility desires or current pregnancy (36.0% [41/114]), a desire for longer ART use by the partner living with HIV (15% [17/114]), or unsuppressed virus (12% [14/114]). For HIV-negative partners who did not use PrEP (n=28), 89.3% had partners living with HIV who initiated ART. Only 3 HIV-negative partners (0.3%) did not have protection from either PrEP or their partner’s ART use during follow up. Among the 734 HIV-negative participants who discontinued PrEP, 35 (4.8%) re-started PrEP before the end of the 2-year follow up period due to reasons that included fertility desires (n=14), concerns about ART use and/or viral load in the partner living with HIV (n=5), resolution of an adverse event (n=4), and new sexual partnerships (n=3).

**Figure 3.**
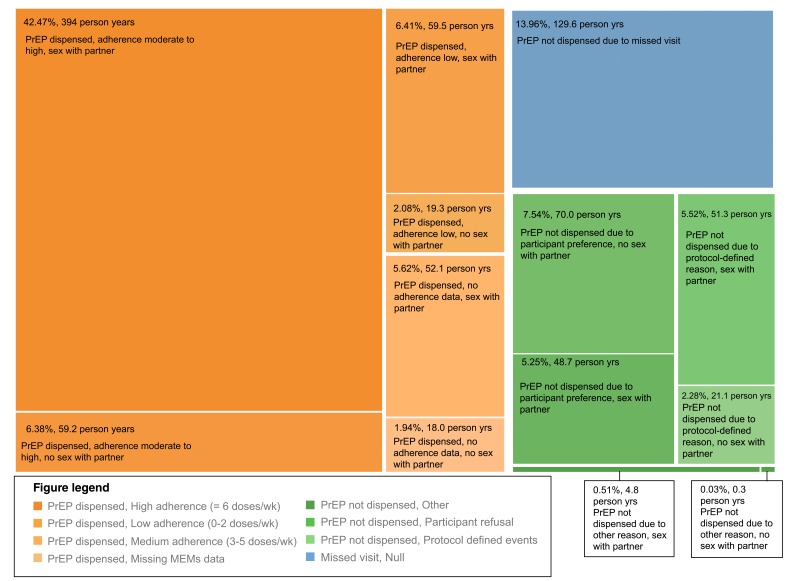
PrEP use by HIV-negative partners from the time of study enrollment through 6 months of ART use by HIV infected partners.

During the follow up period prior to the partner living with HIV having used ART for 6 months (comprising 928.2 person-years), HIV-negative partners were dispensed PrEP for 602.4 person-years (64.9%, Figure 3, orange sections). When PrEP was not dispensed ( Figure 3, green sections), 46.6% included time when no sex with the study partner living with HIV was reported. Of the remaining time without PrEP, 26.1% of time without PrEP was due to a protocol-defined event (pregnancy, abnormal serum creatinine measurement, etc.) and 24.8% was due to participant decision. For 129.6 person-years (14.0% of all study time), participants missed study visits and were unable to be offered PrEP.

*PrEP adherence.* Among 140 participants randomly selected for TFV quantification at 607 visits following PrEP dispensation, 81% of samples had TFV detected (81% from women and 81% from men). MEMS data also were consistent with high adherence: 71% (72% from women and 68% from men) of visits had ≥80% of expected doses taken since the prior visit and 85% (84% from women and 85% from men) had ≥50% taken. Based on pharmacy pill counts, 87% (86% of women and 87% of men) and 96% (95% of women and 97% of men) of bottles had ≥80% and ≥50% of expected doses taken, respectively.

*HIV incidence and intervention effectiveness.* There were 18 seroconversions, including 4 incident seroconversions and 14 determined to have been infected at enrollment. Based on the 4 incident seroconversions, the observed incidence rate was 0.24 per 100 person-years ( Table 2). Using bootstrapping methods with the placebo arm of the Partners PrEP Study, we estimated that 80.7 incident infections were expected in the absence of our intervention, resulting in an estimated incidence of 4.75 per 100 person-years. The incidence rate ratio was 0.05 (95% confidence interval [CI] 0=0.02-0.14) for an intervention effectiveness of 95% (95% CI: 86-98%). The intervention was highly effective in all subgroups examined including women (effectiveness=93%, p<0.0001), HIV-negative partners aged <25 (effectiveness=94%, p<0.0001), and among couples with HIV positive partners having HIV RNA ≥50,000 copies/ml at baseline (effectiveness=95%, p<0.0001).

**Table 2.** Expected versus observed HIV incidence.

	Expectation from Partners PrEP Study *		Observed from Partners Demonstration Project		Incidence rate ratio (95% CI) p-value	Effectiveness (95% CI)
	N incident infections/ N years follow up	Incidence **	N incident infections/ N years follow up	Incidence **		
Overall incidence	80.7/1700.2	4.75	4/1682.3	0.24	0.05 (0.02, 0.14) p<0.0001	95% (86–98%)
By gender						
Women	42.0/553.0	7.60	3/560.4	0.54	0.07 (0.02, 0.23) p<0.0001	93% (77–98%)
Men	41.1/1144.6	3.59	1/1121.9	0.09	0.03 (0.00, 0.18) p<0.0001	97% (82–100%)
By age category						
HIV-negative partner <25 years old	17.1/344.7	4.97	1/332.0	0.30	0.06 (0.01, 0.46) p<0.0001	94% (54–99%)
HIV-negative partner ≥25 years old	62.7/1357.4	4.62	3/1350.3	0.22	0.05 (0.02, 0.15) p<0.0001	95% (85–98%)
By HIV RNA category						
HIV-positive partner HIV RNA ≥50,000 copies/ml	39.3/674.0	5.84	2/707.3	0.28	0.05 (0.01, 0.20) p<0.0001	95% (80–99%)
HIV-positive partner HIV RNA <50,000 copies/ml	41.4/1024.9	4.04	2/975.0	0.21	0.05 (0.01, 0.21) p<0.0001	95% (79–99%)

*The number of expected seroconversions and person-years do not sum precisely to the overall totals because each subgroup estimate is drawn from a separate bootstrapped counterfactual cohort model. **per 100 person-years

Of the 4 individuals with incident HIV infection, none had TFV detected in plasma samples. The partners of three of these individuals were virally suppressed at the time when HIV was detected in the initially HIV-negative partner, and all initially HIV-negative partners reported other sexual partners in addition to their study partner. Thus, partners who were not enrolled in the study (i.e., new or additional partners, whose HIV serostatus was unknown to the study) likely transmitted these infections. For the individual with incident HIV infection whose study partner had quantifiable HIV RNA when HIV was detected, analysis of the *pol* gene sequences from both partners revealed a likely linkage between two sequences. For this woman, her male partner had not yet initiated ART, as his CD4 count of 515 cells/mm ^3^ did not qualify for initiation during the time when Ugandan ART guidelines required CD4 <350 cells/mL. None of the 4 people with incident infection had mutations conferring resistance to TDF or FTC. Five ^5^ of the 14 participants determined to have been infected with HIV at enrollment had an M184V mutation consistent with resistance to FTC.

**Figure 4.**
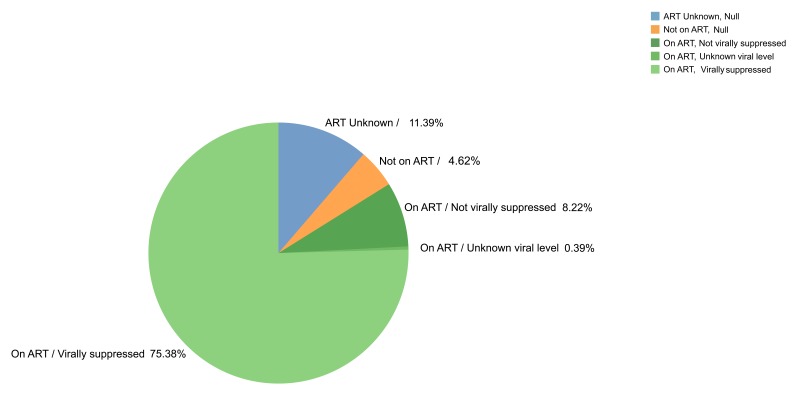
ART status within each couple at the end of the study follow-up.

*HIV protection at study end.* After two years of follow up, 75.4% of couples had a partner living with HIV whose viral load was documented as suppressed and 8.5% had ART use by the partner living with HIV but did not have viral suppression or had unknown viral load levels ( Figure 4). For 4.6% of couples (n=46 couples), ART was not being used by the partner living with HIV, including 20 couples who reported sex together in the past 3 months (15 with 100% condom use and 5 with some condomless sex).

## Discussion

In this open-label evaluation of PrEP for HIV-negative partners within HIV serodiscordant couples, PrEP uptake and adherence were high and incident HIV was virtually eliminated. For 1010 HIV-negative persons followed for 2 years, PrEP was dispensed for 12 months on average. Since the beginning of our evaluation, ART guidelines have become more inclusive and ART use is more standard for all people living with HIV. These changes are likely to result in a shorter average duration of PrEP use since the time between HIV diagnosis and ART initiation and viral suppression is likely shorter for partners living with HIV. When PrEP was not used, a majority of people had HIV protection by their partner’s ART use and viral suppression or had dissolved their partnership with their partner known to be living with HIV.

Our model of PrEP delivery was to introduce PrEP into couples-based HIV prevention and to recommend PrEP discontinuation once the partner living with HIV initiated and sustained ART. Almost all of the HIV-negative partners initiated PrEP and after two years of follow up, 75% of couples could rely on ART use and viral suppression in the partner living with HIV for protection against HIV transmission. Throughout the two-year follow up period, about half of the HIV-negative participants followed the PrEP strategy as our protocol intended, with PrEP used until their partner living with HIV had used ART for 6 months. Others used PrEP for longer or shorter periods overlapping with ART. These metrics demonstrate the feasibility for potentially implementing this strategy in East Africa as well as the ability to integrate PrEP into an existing clinical structure.

When PrEP was dispensed, adherence was moderate to high affording early protection from HIV acquisition and establishing a sustainable adherence behavior. Demonstration projects have shown greater adherence to PrEP than was observed in the clinical trials, potentially due to messages clearly describing the efficacy of PrEP and because they are often implemented in public clinics, which are more familiar than clinical trial settings. Importantly, adherence appears to align with HIV risk. Incident HIV infections occurred in our study in the absence of PrEP use and primarily outside of the study partnerships. These instances highlight opportunities to expand PrEP messaging to incorporate risk within newly forming relationships and with casual partners or transactional partners. For young people, especially those who are not yet married, messages need to be realistic and recognize the dynamic nature of relationships in order to encourage open communication with counselors and counseling grounded in realistic prevention strategies.

This intervention gave HIV-negative persons with partners living with HIV a means for engaging with healthcare and a primary prevention strategy. By engaging as couples, the partner living with HIV gained familiarity with clinical care and antiretroviral use, enhancing their knowledge of ART programs with client trust and abilities to initiate ART faster ^13^. This integrated approach provides support to each partner within the couple as well as the couple as a unit. Without PrEP, the HIV-negative partner has to rely only on condom use for primary prevention until the eventual identification of HIV, ART initiation, and sustained ART use with viral suppression. Condom use within marriage has been reported to be difficult due to cultural norms that discourage condom use within marriage, desires for pregnancy, and difficulties internalizing and coping with discordancy ^14^.

Additional benefits of this intervention include improved relationship stability and strengthening communication and negotiation skills between couples that have been reported elsewhere ^15, 16^. These benefits foster opportunities for couples to discuss their sexual behavior, plans for pregnancy, concerns about HIV risk, and other topics that are sensitive but important for reducing HIV risk ^17^. By designing this study under an implementation science framework, we had an opportunity to refine standardized messages for couples about biomedical HIV prevention and pilot add-on components that would increase efficiencies in intervention delivery, such as HIV self-testing, less frequent monitoring of kidney function, and adherence support through text messages ^10, 18– 20^.

Within this study, microcosting analysis suggests cost-effectiveness, with the largest portion of costs owing to purchasing medication and laboratory monitoring ^21^. Most PrEP users in this project needed PrEP for a limited time until ART use and viral suppression could be the primary mode of HIV protection. This attribute contributes to the cost-effectiveness of this delivery strategy but does not fully account for the frequency of re-starting PrEP during follow up beyond 2 years. Future evaluations within sustainable delivery programs will provide estimates of how frequently PrEP is re-started and the cost implications. As PrEP delivery programs are scaled up, identifying opportunities to improve efficiency from the patient and provider perspectives will reduce costs and potentially enhance effectiveness.

We measured adherence with MEMS caps and laboratory markers that are research tools and unlikely suitable for public clinics given their cost and logistical requirements (shipping samples to the US/central laboratory, provision of MEMs to each participant, etc.). However, programmatic scale up of HIV viral load testing is being implemented and can be used to guide counseling about ART adherence and prevention of transmission and to reinforce messages about PrEP adherence when viral load is not suppressed. In addition, we provided participants with a small reimbursement as a token of appreciation for their participation in research procedures and we cannot tease apart the degree to which this influenced participant retention in the project. A final limitation is that our model does not consider additional HIV risk factors, such as the frequency of condomless sex. During follow up, couples in the Partners Demonstration Project reported condomless sex more frequently than those in the comparison study and thus this group is likely to have higher risk.

PrEP delivery to HIV-negative individuals with partners known to be living with HIV was highly effective. HIV-negative participants used PrEP and most discontinued in parallel with sustained ART use by their partner living with HIV, transferring their HIV protection to their partner’s ART use. Offering PrEP as a feature of existing ART programs must be done via messages and materials that are tailored for couples in order to reach members of HIV serodiscordant couples. While multiple venues for PrEP provision are likely needed in order to tailor services to different subpopulations, providing PrEP through ART clinics takes advantage of existing infrastructure, has benefits for both partners within HIV serodiscordant couples, and is feasible to roll out on a national level.

## Data availability

Data are available upon request to the authors’ research center, by emailing icrc@uw.edu with a concept sheet stating the objectives of the analysis and variables desired.

## Partners Demonstration Project Team

**Coordinating Center (University of Washington) and collaborating investigators (Harvard Medical School, Johns Hopkins University, Massachusetts General Hospital):** Jared Baeten (protocol chair), Connie Celum (protocol co-chair), Renee Heffron (project director), Deborah Donnell (statistician), Ruanne Barnabas, Jessica Haberer, Harald Haugen, Craig Hendrix, Lara Kidoguchi, Mark Marzinke, Susan Morrison, Jennifer Morton, Norma Ware, Monique Wyatt

## Project sites

Kabwohe, Uganda (Kabwohe Clinical Research Centre): Stephen Asiimwe, Edna Tindimwebwa

Kampala, Uganda (Makerere University): Elly Katabira, Nulu Bulya

Kisumu, Kenya (Kenya Medical Research Institute): Elizabeth Bukusi, Josephine Odoyo

Thika, Kenya (Kenya Medical Research Institute, University of Washington): Nelly Rwamba Mugo, Kenneth Ngure

Data management: DF/Net Research

## Supplementary material

**Supplementary File 1: Completed CONSORT checklist.**

**Supplementary File 2: Completed CONSORT flow diagram.**
