# Supplementary figures and images for: Pre-exposure prophylaxis for HIV-negative persons with partners living with HIV: uptake, use, and effectiveness in an open-label demonstration project in East Africa
Source: Gates Open Res. 2018 Jan 30;1:3. Originally published 2017 Nov 6. [Version 2] doi: 10.12688/gatesopenres.12752.2 (PMC5757790; doi:10.12688/gatesopenres.12752.2)

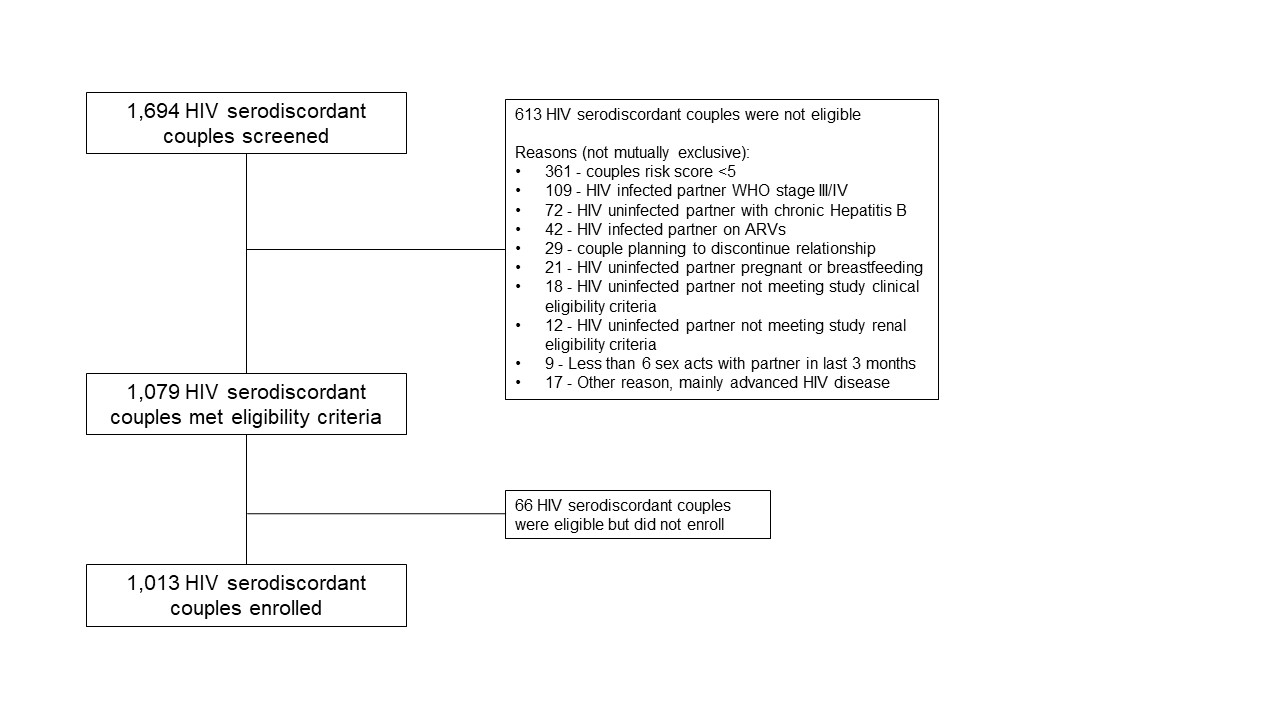

Supplement: Supplementary file 2 [file gatesopenres-1-13860-s0001.tgz › b80b2108-a4e5-43d1-af54-8f25d9e2c6b3.JPG]
