# Supplementary material for: Pre-exposure prophylaxis for HIV-negative persons with partners living with HIV: uptake, use, and effectiveness in an open-label demonstration project in East Africa
Source: Gates Open Res. 2018 Jan 30;1:3. Originally published 2017 Nov 6. [Version 2] doi: 10.12688/gatesopenres.12752.2 (PMC5757790; doi:10.12688/gatesopenres.12752.2)
Supplement: Peer review history

# Peer review: Referee response for version 2

**DOI:** 10.21956/gatesopenres.13860.r26233

**Published:** 2018-01-31

**Authors:** George W. Rutherford

**Affiliations:** 1 Department of Epidemiology & Biostatistics, University of California, San Francisco, San Francisco, CA, USA

No new comments. All my suggestions have been incorporated. Thanks.

I have read this submission. I believe that I have an appropriate level of expertise to confirm that it is of an acceptable scientific standard.

# Peer review: Referee response for version 1

**DOI:** 10.21956/gatesopenres.13812.r26101

**Published:** 2017-12-04

**Authors:** John Idoko, Morenike Ukpong

**Affiliations:** 1 National Agency for the Control of AIDS, Abuja, Nigeria; 2 College of Health, Obafemi Awolowo University, Ile-Ife, Nigeria

The article reports on the outcome of one of two global PrEP demonstration projects with HIV-1 sero-discordant couples as intervention recipients. The study is therefore a significant contribution to understanding uptake and use of PrEP in this target population in additional to the additional finding on PrEP effectiveness for the population. It is indeed a very useful addition to the literature already generated by the Partners Demonstration Project Team. A few issues however:

**1. Title:** Appropriate as  the study had focused on the themes – uptake, use and effectivenss of PrEP in their study. The study is very relevant, given the impact PrEP can make on combination prevention. 

**2. Abstract: **It will be helpful to have the study objective defined in the introduction of the abstract. The methodology does not state how the uptake of PrEP was assessed and this seems to be one of the objectives of the study. 

**3. Introduction: **Detailed. The introduction considers the full context of the research topic and has gone to describe the impact of PrEP in serodiscordant partners when the HIV positive partner is not taking ART or in the short term (6 months) before the expected viral suppression. 

**4. Methods:** The study noted: ‘whenever possible, operations for the intervention were designed to mirror implementation strategies used in public clinics so as to develop a scalable delivery approach’. This statement is critical. I suggest the authors identify implementation practices that are not routine public clinic practices that may have had impact on the observed results. For example, in real life, will clients be screened for hepatitis B virus before being placed on PrEP? Where clients provided transport reimbursements for this study and if yes, how did that affect adherence to study routine that may otherwise had impacted on missed appointments, loss to follow up (currently the study shows 14% of study time)? How were clients monitored and followed up for appointments for this project which may otherwise not happen in the routine public clinic practice? Can the quality of adherence messaging for this project be replicable in the public clinics? What will be the impact of these extra efforts (if applicable) in a scalable approach? We suggest the team reports on those project implementation strategies that are likely not going to be replicated in the public clinic practices that may impact significantly on the uptake, use and effectiveness of PrEP for sero-discordant couples. 

 The authors noted that HIV RNA tests were conducted at study enrolment and at 6 months intervals. “HIV negative partners were encouraged to discontinue PrEP once their study partners used ART for six months”. The viral load suppression is key to the effectiveness of PrEP and hence should be used to gage discontinuation at 6 months. Yet, these results were not used to guide decisions about discontinuation of PrEP at six months for clients. “HIV RNA results could be used to identify the potential for non-adherence to ART”. Why will the authors invest so much in the use of HIV RNA to identify non-adherence when they are already using other measures including self reporting, MEMS caps and blood levels of TDF? Particularly when they state at the discussion following microcosting, the largest costs were from drugs and laboratory monitoring which will have HIV RNA testing as a major component cost. What then was the justification for doing those tests for this study? What is the ethical implication of doing those test and yet not using then as guide to make decisions about going off PrEP for this study? What is the ethical justification of taking this decisions when the study had made some other implementation decisions that were not routine practices in a public health clinic in Kenya and Uganda? The justification for this decision remains unclear to us and would give an impression that HIV RNA is not an important indicator in deciding when to stop PrEP. The new ART guidelines recommend viral load testing and it is key to achieving the 3 ^rd^ 90. Please do justify the reason for conduct of routine HIV RNA tests and yet not using the results for PrEP discontinuation decision making for this study.

 The authors note that *If available when PrEP discontinuation was considered, HIV RNA results could be used to identify the potential for non-adherence to ART. *Can the authors provide clarity about the term ‘ could’ in this statement? How can an algorithm of practice be developed when there are critical elements of a PrEP management process that is subjective in its implementation. It will be good to have some clarification on this practice by the PrEP team. We noticed the results showed that 12% of 114 clients who continued PrEP use beyond 6 months did so because of un-supressed viral load. How was this determined if the HIV RNA results was not routinely used to guide the counselling session? Just curious and need clarity about this.

 How frequently was PrEP dispensed for this study? How was PrEP used –daily or coitally dependent? How frequently were HIV tests conducted? It is important that these details are reflected in the manuscript. Where condoms provided to study participants and if so, how frequently?

 The authors have a section dedicated to describe how PrEP adherence was measured. Adherence seems to be the buss word in the field. However, We think the authors captured what they assessed better when they used the word ‘use’. We think what the authors tried to assess was truly use. Adherence implies sticking to a routine schedule. The clients were expected to use PrEP everyday not at a specific time schedule right?

**Results**

 Extremely curious why WHO Stage >2 was a reason for non-eligiblity of the HIV negative partner for enrolment on PrEP. How will such an enrolment stategy play out in the routine practice in a public clinic? Why should the HIV positive partners with WHO Stage >2 be screened out of the study? Is this not some form of bias?

 We suggest this section be divided into section to help readers identify how the results are reflective of the objectives. Sections to show results on PrEP uptake, use and effectiveness will be great.

 It would be good to highlight the profile (characteristics) of the 3% (n=28) of eligible participants who did not take up PrEP.

 The study reports on those who used PrEP for more than 6 months and why. It will be good to know precisely how many people continued the use of PrEP  6-12 months and for 12-18 months. 12 months is a critical period for this study as this was stipulated in the study protocol as the time for study product access. The study highlighted the number that continued beyond 9 months.

 Any gender differences in the profile of PrEP uptake and use? This is a very important study analysis that is missing from this manuscript. Very, very, very important especially in the context of PrEP use in a community where women are vulnerable in many ways.

**Discussion**

 The last sentence in the first paragraph states that *: When PrEP was not used, a majority of people had HIV protection by their partner’s ART use and viral suppression or had dissolved their partnership with their partner known to be living with HIV. *There were only 3 clients that changed sex partners. This does not fall into a category of majority.

 The last sentence of the second paragraph states that: *These metrics demonstrate the feasibility of implementing this strategy in East Africa as well as the ability to integrate PrEP into an existing clinical structure. *This study DOES NOT demonstrate the ability to integrate PrEp into an existing clinical structure. The study did not measure any hospital related parameters that may impact positively or negatively on PrEP uptake, use and intake and so that conclusion is spurious. We suggest this be deleted or modified.

 Column 2, paragraphs 3 that alludes to cost effectiveness. Most of the costs went to drugs and tests and we presume measuring viral loads must have been a capital cost. What is the cost-effectiveness of such intervention if it is implemented in public clinics where viral loads are done not for deciding the point of stopping PrEP but monitoring adherence, particularly when there are other measures for adherence.

 A sentence in the last paragraph reads - *Offering PrEP as a feature of existing ART programs must be done via messages and materials that are tailored for couples. *We suggest that the paragraph be re-written to acknowledge that the suggestion is for sero-discordant couples.

 For the PrEP demonstration project in Nigeria, we had identified a number of couples that do not want to access PrEP and TasP by coming to the clinic together as couples but as individuals. Did the study encounter such experience? Are there study limitations?

 Strong points in the study

- Short term PrEP for 6 months
- High level of adherence
- Very few incident infections
- Improved couple relationships in sexual behaviour
- Implementation science strategy      

Weak ponts in the study

- Needs more detailed description of PrEP
- More details of the implementation science strategy in the methodology
- Poor use of HIV RNA to identify point of viral suppression in the HIV positive partner.

We have read this submission. We believe that we have an appropriate level of expertise to confirm that it is of an acceptable scientific standard.

# Response

**Published:** 2018-01-29

**Authors:** Renee Heffron

**We are grateful for the reviewers’ efficient and generally favorable review. We have responded to each point below.**

**1. Title:** Appropriate as  the study had focused on the themes – uptake, use and effectivenss of PrEP in their study. The study is very relevant, given the impact PrEP can make on combination prevention. 

**Thank you for your favorable comment.**

**2. Abstract: **It will be helpful to have the study objective defined in the introduction of the abstract. The methodology does not state how the uptake of PrEP was assessed and this seems to be one of the objectives of the study. 

**We have added a sentence to the Introduction to describe the manuscript objective and a phrase to the Methods describe how the uptake of PrEP was assessed. Due to the limit on abstract size, we have had to remove ~25 words in order to maintain these additions.**

**3. Introduction: **Detailed. The introduction considers the full context of the research topic and has gone to describe the impact of PrEP in serodiscordant partners when the HIV positive partner is not taking ART or in the short term (6 months) before the expected viral suppression. 

**Thank you for your favorable comment.**

**4. Methods:** The study noted: ‘whenever possible, operations for the intervention were designed to mirror implementation strategies used in public clinics so as to develop a scalable delivery approach’. This statement is critical. I suggest the authors identify implementation practices that are not routine public clinic practices that may have had impact on the observed results. For example, in real life, will clients be screened for hepatitis B virus before being placed on PrEP? Where clients provided transport reimbursements for this study and if yes, how did that affect adherence to study routine that may otherwise had impacted on missed appointments, loss to follow up (currently the study shows 14% of study time)? How were clients monitored and followed up for appointments for this project which may otherwise not happen in the routine public clinic practice? Can the quality of adherence messaging for this project be replicable in the public clinics? What will be the impact of these extra efforts (if applicable) in a scalable approach? We suggest the team reports on those project implementation strategies that are likely not going to be replicated in the public clinic practices that may impact significantly on the uptake, use and effectiveness of PrEP for sero-discordant couples. 

**This is an important point and we have added some examples to the methods of how we mirrored public clinics processes. We have also added one point to the discussion about participant reimbursement that was given as a token of appreciation for participation in the research procedures that were part of the intervention. Below, we have provided a few more details about the specific elements that the reviewer mentioned.**

**Hepatitis B – Globally, national guidelines are using various approaches to their recommendation about Hepatitis B testing prior to PrEP initiation. Guidelines in South Africa, for example, require Hepatitis B testing and resulting prior to PrEP initiation. In Kenya, however, guidelines suggest Hepatitis B antigen testing but PrEP can be initiated without this testing if laboratory facilities are not available and/or the testing is not feasible. Thus, our method aligns with public health approaches to date.**

**Reimbursement – We provided reimbursement to participants because some of the procedures (e.g. consenting and occasional blood draws) were research procedures and would go beyond the public clinic approach. We worked with ethics committees to minimize the amounts of reimbursement as much as was reasonable so as not to be an undue incentive for retention or PrEP use.**

**Participant monitoring and follow up – We followed systems at local ART clinics very closely when we designed our follow up procedures. Our primary method for tracking participants between study visits was by making phone calls or sending confidential text messages. To maximize participation at annual visits, people who had difficulty attending appointments were asked to gather at a central location near to their home and provided with transportation to the clinic.**

**Adherence counseling – We have published a separate manuscript (reference #10) that details our approach to counseling about this integrated PrEP and ART strategy, including the key messages for adherence counseling. By publishing this information, we intended to make the key counseling messages available and replicable in public clinics.**

**Public clinic practices – In an ongoing PrEP rollout project in Kenya in 24 public ART clinics, this strategy of integrating PrEP into HIV treatment services is being replicated (clinicaltrials.gov #NCT03052010).**

5. The authors noted that HIV RNA tests were conducted at study enrolment and at 6 months intervals. “HIV negative partners were encouraged to discontinue PrEP once their study partners used ART for six months”. The viral load suppression is key to the effectiveness of PrEP and hence should be used to gage discontinuation at 6 months. Yet, these results were not used to guide decisions about discontinuation of PrEP at six months for clients. “HIV RNA results could be used to identify the potential for non-adherence to ART”. Why will the authors invest so much in the use of HIV RNA to identify non-adherence when they are already using other measures including self reporting, MEMS caps and blood levels of TDF? Particularly when they state at the discussion following microcosting, the largest costs were from drugs and laboratory monitoring which will have HIV RNA testing as a major component cost. What then was the justification for doing those tests for this study? What is the ethical implication of doing those test and yet not using then as guide to make decisions about going off PrEP for this study? What is the ethical justification of taking this decisions when the study had made some other implementation decisions that were not routine practices in a public health clinic in Kenya and Uganda? The justification for this decision remains unclear to us and would give an impression that HIV RNA is not an important indicator in deciding when to stop PrEP. The new ART guidelines recommend viral load testing and it is key to achieving the 3 ^rd^ 90. Please do justify the reason for conduct of routine HIV RNA tests and yet not using the results for PrEP discontinuation decision making for this study.

**HIV RNA testing was conducted so we would have the strongest measure of ART adherence as possible for our research outcomes. We considered this testing to be a research procedure, however, since it was not available routinely in public clinics at the time of our study (2012-2016) and couldn’t necessarily be a procedure replicated in public clinics. At this time, clinics were generally only referring for HIV RNA when virologic failure was suspected. Even today, HIV RNA testing is not routine and universal in public ART delivery in Kenya and Uganda. Thus, as a research procedure, we scheduled HIV RNA testing on a 6-monthly basis which was operationally more simple than aligning with individual ART initiation schedules. Since many people did not have a relevant HIV RNA result that was available during counseling about ART adherence and/or PrEP discontinuation, we did not *require* HIV RNA results to guide discussion about PrEP discontinuation. But of course, any relevant HIV RNA result could be used for counseling if it was available. We have explained that in the following sentence: *“*** ***If available when PrEP discontinuation was considered, HIV RNA results could be used to identify the potential for non-adherence to ART; however, testing was conducted on a 6-monthly schedule timed to study start, not the ART initiation schedule, and testing may not have aligned with ART initiation.”***

**The laboratory testing that is discussed in relation to the microcosting is Hepatitis B and creatinine, which would be additional laboratory tests pertaining to PrEP that would need to be added into an existing HIV treatment program. The full paper on microcosting is cited in the references (#21).**

6. The authors note that *If available when PrEP discontinuation was considered, HIV RNA results could be used to identify the potential for non-adherence to ART. *Can the authors provide clarity about the term ‘ could’ in this statement? How can an algorithm of practice be developed when there are critical elements of a PrEP management process that is subjective in its implementation. It will be good to have some clarification on this practice by the PrEP team. We noticed the results showed that 12% of 114 clients who continued PrEP use beyond 6 months did so because of un-supressed viral load. How was this determined if the HIV RNA results was not routinely used to guide the counselling session? Just curious and need clarity about this.

**These data on PrEP continuation due to unsuppressed viral load were collected from the participant following their counseling session with study clinicians and counselors. For times when HIV RNA was conducted following ART initiation and results were available for discussion with the HIV-negative member and showed unsuppressed virus, some participants chose to continue PrEP because of these laboratory results. When HIV RNA was not available and a participant suspected suboptimal ART adherence by their partner, the participant could also choose to continue PrEP.**

7. How frequently was PrEP dispensed for this study? How was PrEP used –daily or coitally dependent? How frequently were HIV tests conducted? It is important that these details are reflected in the manuscript. Where condoms provided to study participants and if so, how frequently?

**We have ensured that all of these details are now clearly presented in the manuscript. PrEP was dispensed at each study visit – enrollment, 1 month later, 2 months later, and every 3 months after that – with a daily dosing strategy. HIV testing was conducted at each study visit and always before PrEP was prescribed.**

8. The authors have a section dedicated to describe how PrEP adherence was measured. Adherence seems to be the buss word in the field. However, We think the authors captured what they assessed better when they used the word ‘use’. We think what the authors tried to assess was truly use. Adherence implies sticking to a routine schedule. The clients were expected to use PrEP everyday not at a specific time schedule right?

**We thank the reviewer for this discussion. We generally use “adherence” as a more technical term referring to compliance with the daily dosing routine. We used the word “use” as a more colloquial and approachable term to encompass the ideas of PrEP initiation and adherence. Our intention was to look at PrEP adherence and patterns describing PrEP uptake, discontinuation, and re-initiation – “use” is a way to encompass all of this description of PrEP into a single word.**

**Results**

9. Extremely curious why WHO Stage >2 was a reason for non-eligiblity of the HIV negative partner for enrolment on PrEP. How will such an enrolment stategy play out in the routine practice in a public clinic? Why should the HIV positive partners with WHO Stage >2 be screened out of the study? Is this not some form of bias?

**Since WHO stage >2 was a criteria for ART initiation, we wanted to be sure that people with WHO stage >2 sought immediate engagement with a regular ART clinic and were not caught up in any research procedures that might delay their ART initiation (e.g. additional screening visits). We have explained this in the methods saying that *“couples were excluded if the HIV-positive partner had WHO stage III or IV HIV disease conditions that indicated immediate need for ART.”***

10. We suggest this section be divided into section to help readers identify how the results are reflective of the objectives. Sections to show results on PrEP uptake, use and effectiveness will be great.

**We have added section headers as suggested.**

11. It would be good to highlight the profile (characteristics) of the 3% (n=28) of eligible participants who did not take up PrEP.

**The number of people in this group are so few that we are unable to identify distinct characteristics of the people not initiating PrEP.**

12. The study reports on those who used PrEP for more than 6 months and why. It will be good to know precisely how many people continued the use of PrEP  6-12 months and for 12-18 months. 12 months is a critical period for this study as this was stipulated in the study protocol as the time for study product access. The study highlighted the number that continued beyond 9 months.

**The study availed PrEP access to participants for a 2-year period. We reported on 6 months specifically because of our guidance to discontinue PrEP once ART use had been sustained by partners living with HIV for at least 6 months. We have reported the median duration of PrEP use as 12 months with an interquartile range of 6-18 months.**

13. Any gender differences in the profile of PrEP uptake and use? This is a very important study analysis that is missing from this manuscript. Very, very, very important especially in the context of PrEP use in a community where women are vulnerable in many ways.

**We have added gender-specific details to the manuscript with regards to PrEP initiation and adherence.**

**Discussion**

14. The last sentence in the first paragraph states that *: When PrEP was not used, a majority of people had HIV protection by their partner’s ART use and viral suppression or had dissolved their partnership with their partner known to be living with HIV. *There were only 3 clients that changed sex partners. This does not fall into a category of majority.

**With this sentence, we were referring to the ~10% of person time when PrEP was not dispensed and there was no sex reported with the study partner. These person years represent approximately half of the time when PrEP was not dispensed and study visits were attended (see Figure 3).**

15. The last sentence of the second paragraph states that: *These metrics demonstrate the feasibility of implementing this strategy in East Africa as well as the ability to integrate PrEP into an existing clinical structure. *This study DOES NOT demonstrate the ability to integrate PrEp into an existing clinical structure. The study did not measure any hospital related parameters that may impact positively or negatively on PrEP uptake, use and intake and so that conclusion is spurious. We suggest this be deleted or modified.

**We have modified the sentence. We now say that, *“These metrics demonstrate the feasibility for potentially implementing this strategy in East Africa as well as the ability to integrate PrEP into an existing clinical structure.”***

16. Column 2, paragraphs 3 that alludes to cost effectiveness. Most of the costs went to drugs and tests and we presume measuring viral loads must have been a capital cost. What is the cost-effectiveness of such intervention if it is implemented in public clinics where viral loads are done not for deciding the point of stopping PrEP but monitoring adherence, particularly when there are other measures for adherence.

**The full data on microcosting that was conducted within this study can be found by reading reference #21 (Ying et al.). An additional manuscript with details of the costs for implementing this intervention in Kenya using Ministry of Health standards is under review.**

17. A sentence in the last paragraph reads - *Offering PrEP as a feature of existing ART programs must be done via messages and materials that are tailored for couples. *We suggest that the paragraph be re-written to acknowledge that the suggestion is for sero-discordant couples.

**We have added to the sentence to reflect this suggestion.**

18. For the PrEP demonstration project in Nigeria, we had identified a number of couples that do not want to access PrEP and TasP by coming to the clinic together as couples but as individuals. Did the study encounter such experience? Are there study limitations?

**In this demonstration project, we only screened people on the basis of being part of a couple.**

# Peer review: Referee response for version 1

**DOI:** 10.21956/gatesopenres.13812.r26099

**Published:** 2017-11-29

**Authors:** George W. Rutherford

**Affiliations:** 1 Department of Epidemiology & Biostatistics, University of California, San Francisco, San Francisco, CA, USA

This is a well written manuscript that describes results from a well-designed and executed demonstration project of tenofovir-emtricitabrine (TDF-FTC) pre-exposure prophylaxis of HIV-seronegative sexual partners of patients recently diagnosed with HIV infection. The intervention was delivered during the pre-antiretroviral therapy (ART) phase of care and/or for the first six months of ART. The investigators compared the observed HIV incidence in uninfected partners (0.24 per 100 person years) with that observed during the Partners’ PrEP Study (4.75 per 100 person years). The results are compelling and suggest an important role for short-term PrEP in serodiscordant couples in which the infected partner is beginning ART. This is an important paper that will likely change policy.

 Some specific comments:

 Page    Para    Comment

 5          2          I suggest adding the word “any” before “condomless sex” in the text. Is there any data on what proportion of acts of intercourse among these discordant couples that were unprotected by condoms?

 5          2          At the bottom of the first Results paragraph, the second to last sentence reads, “Ninety-seven (97%) of HIV-negative participants initiated PrEP…” This should be, I believe, “Nine hundred eighty-two”.

 6                      I found Figure 2 a little difficult to get through. It might be helpful to use terms like “HIV-positive partner” and be clear that you’re talking about the uninfected partners in the third column of boxes. These are minor changes, but I think they’d enhance readers’ quicker understanding.

 6          3          This comment may be more appropriate for the Methods, but how did the authors deal with protection afforded by condoms and abstinence? It would appear to me that these factors were not taken into account in the incidence calculations either from this study or the Partners’ PrEP Study. I think this deserves some mention in the Discussion although I don’t think it changes the overall intervention effectiveness calculated.

 7                      I realize the color coding for Figure 3 appears in the text. I suggest that the authors also include a legend with the figure. I would also suggest indicating in which box the single genetically linked transmission took place (I’m assuming it’s the 6.41% (PrEP dispensed, adherence low, sex with partner).

I have read this submission. I believe that I have an appropriate level of expertise to confirm that it is of an acceptable scientific standard.

# Response

**Published:** 2018-01-29

**Authors:** Renee Heffron

**We are grateful for the review’s efficient and favorable review. We have responded to each point below.**

1. I suggest adding the word “any” before “condomless sex” in the text. Is there any data on what proportion of acts of intercourse among these discordant couples that were unprotected by condoms?

**We have added “any” to the text, as suggested. Data on the frequency of condomless sex are presented in Table 1 where we describe that 65% of couples reported condomless sex during the 30 days prior to study enrollment. We have also added a detail to the Results that couples reported condomless sex at 41.2% of study visit.**

2. At the bottom of the first Results paragraph, the second to last sentence reads, “Ninety-seven (97%) of HIV-negative participants initiated PrEP…” This should be, I believe, “Nine hundred eighty-two”.

**We have updated the sentence to read “Nine hundred eighty-two.” The “ninety-seven” was meant to spell out the percentage in the parentheses since it is at the start of the sentence but we agree that this is confusing.**

3. I found Figure 2 a little difficult to get through. It might be helpful to use terms like “HIV-positive partner” and be clear that you’re talking about the uninfected partners in the third column of boxes. These are minor changes, but I think they’d enhance readers’ quicker understanding.

**We have updated the figure and the figure title to use more comprehensive descriptions in the boxes and made sure that all the descriptions are in the perspective of the HIV-negative partner.**

4. This comment may be more appropriate for the Methods, but how did the authors deal with protection afforded by condoms and abstinence? It would appear to me that these factors were not taken into account in the incidence calculations either from this study or the Partners’ PrEP Study. I think this deserves some mention in the Discussion although I don’t think it changes the overall intervention effectiveness calculated.

**We did not adjust our effectiveness estimates for condom use because we wanted to estimate the level of risk including with the background level of condom use. Condom use at enrollment is a factor within the risk scoring tool that was used to screen couples for the Partners Demonstration Project and to select couples from the Partners PrEP Study for inclusion in the comparison cohort. Thus, condom use at enrolment was somewhat balanced in our effectiveness model. We have also added a sentence to the Discussion to draw this point to the reader’s attention.**

5. I realize the color coding for Figure 3 appears in the text. I suggest that the authors also include a legend with the figure. I would also suggest indicating in which box the single genetically linked transmission took place (I’m assuming it’s the 6.41% (PrEP dispensed, adherence low, sex with partner).

**We have added a legend to the Figure.**
